# Cascade of care for type 2 diabetes mellitus and hypertension in rural Bangladesh: sub-regional mapping of gaps in care

**DOI:** 10.1136/bmjph-2025-004574

**Published:** 2026-09-11

**Authors:** Kristina Bergman, Sanjit Shaha, Joanna Morrison, Naveed Ahmed, Abdul Kuddus, Malini Pires, Tasmin Nahar, Hassan Haghparast-Bidgoli, AK Azad Khan, Kishwar Azad, Edward Fottrell, Carina King

**Affiliations:** 1 Department of Global Public Health, Karolinska Institutet, Stockholm, Sweden; 2 Diabetic Association of Bangladesh, Dhaka, Dhaka District, Bangladesh; 3 Institute for Global Health, University College London, London, UK

**Keywords:** Hypertension, Diabetes Mellitus, Community Health

## Abstract

**Introduction:**

Bangladesh has a high burden of type two diabetes mellitus (T2DM) and hypertension. To better target non-communicable disease (NCD) programmes, populations with unmet care needs must be identified. We describe the unmet care needs for people living with T2DM and hypertension in rural Bangladesh at a subregional level.

**Methods:**

We conducted a secondary analysis of cross-sectional surveys conducted in 2021 and 2022 in Alfadanga Upazila, Faridpur District. Surveys were conducted among random samples of non-pregnant adults aged >30 years, including questions about demographics, NCD diagnoses, care-seeking and treatment. Study staff conducted blood pressure measurements to determine hypertension and 2-hour oral glucose tolerance tests to determine T2DM status. We calculated the prevalence of T2DM and hypertension and the unmet need for care, defined as the prevalence minus the proportion screened (T2DM only), diagnosed, treated and controlled, following the cascade of care (COC). We present the COC for 12 subregional geographical clusters and sex.

**Results:**

1982 participants (62.9% women) were analysed. The prevalence of T2DM was 19.0%, and the unmet need for care was 90.7%. The prevalence of hypertension was 37.9%, and the unmet need for care was 81.4%. For both T2DM and hypertension, women had a higher prevalence of disease, but there was a wider gap in the COC for hypertension, with 22.4% of women achieving good control compared with 10.9% of men. At the subregional level, the prevalence of T2DM ranged from 12.4% to 26.5%, and the unmet need for care from 85.0% to 95.6%. Variations in the prevalence and unmet need for care were also seen for hypertension but with different subregional distribution patterns.

**Conclusions:**

In this rural Bangladesh setting, the unmet care need was high for T2DM and hypertension, with subregional and sex differences. Understanding hyper-local factors driving NCD risks and access to care could support more effective programme delivery.

**WHAT IS ALREADY KNOWN ON THIS TOPIC**

- Variations in type 2 diabetes mellitus (T2DM) and hypertension prevalence and care coverage have been observed at regional, national and subnational levels and between rural and urban settings in South Asia. However, hyper-local variations within a rural Bangladesh context have been less explored.

**WHAT THIS STUDY ADDS**

- This study shows high variation in the prevalence of T2DM and hypertension in rural Bangladesh between small geographical areas. Differences in care coverage and the unmet need for care were observed between these areas and by sex, raising important questions around equity of care access.

**HOW THIS STUDY MIGHT AFFECT RESEARCH, PRACTICE OR POLICY**

- The existence of large subdistrict differences in unmet care need highlights the need for local surveillance systems and routine monitoring data to better direct care services and develop context-specific interventions, which account for sex.

## Introduction

 Type two diabetes mellitus (T2DM) and hypertension (HTN) are two of the most common non-communicable diseases (NCDs), affecting 529 million and 1.28 billion people worldwide, respectively.^1,2^ The main burden of NCDs is in low-income and middle-income countries (LMICs), where 85% of deaths occur.^3^ In South Asia, HTN has increased by 144% from 1990 to 2019,^4^ and T2DM has nearly doubled between 2000 and 2021.^5^ Because of their overlapping risk factor profile, NCDs can co-exist in the same individual (ie, multimorbidity), resulting in increased burden for patients, polypharmacy and the need for a more complex organisation of care.^6^

Bangladesh has experienced rapid urbanisation, with corresponding increases in sedentary lifestyles and consumption of processed foods with higher salt content, which are thought to be driving the country’s increasing prevalence of both T2DM and HTN.^7^ According to the 2022 Bangladesh Demographic Health Survey (BDHS), the national prevalence of T2DM among women aged ≥18 years was 17% and that for men was 15%.^8^ The prevalence of HTN was 23% for women and 17% for men.^8^ Regionally, however, the prevalence of T2DM for women and men ranged from 12% to 23% and from 10% to 20%, respectively, and the corresponding ranges for HTN were 20%–28% and 10%–20%.^8^ A 2019 study reporting survey results from urban and rural Bangladesh showed that 8.4% of adults aged >35 were suffering from multimorbidity,^9^ with HTN and T2DM being the most common.

T2DM and HTN are often asymptomatic for many years, and in healthcare systems without regular screening programmes, diagnosis can be delayed until the development of complications such as blindness or stroke.^10,11^ Early detection and appropriate treatment, including information sharing and advice, can lead to lower rates of complications.^4,10,11^ While T2DM and HTN can be diagnosed using relatively simple procedures, this relies on functioning primary care systems that screen the right people, have the correct equipment and trained staff to make a diagnosis and protocols, and have referral systems in place to initiate and optimise treatment.^12,13^ For many LMICs, the transition from a disease burden consisting mainly of episodic infectious disease and maternal and child illness to NCDs has been rapid, and healthcare systems have struggled to adapt.^13^ As a result, one of the greatest public health challenges relating to T2DM and HTN is the undiagnosed burden, with a third of those with T2DM being aware of their condition and around half for those with HTN.^8,14^

The cascade of care (COC) framework was originally developed to describe the key gaps to effective treatment coverage for HIV and tuberculosis^15^ and has since been adapted to other conditions, including NCDs.^16^ It outlines the sequential steps in care for a condition to be considered effectively treated (eg, awareness of own diagnosis, being on treatment and achieving control), with the gap between the disease prevalence and control representing an unmet need for care. A 2024 scoping review reported 128 studies from 40 different countries that had used the COC framework for HTN and T2DM.^17^ Most of these were conducted in high-income countries, for a single disease, and used a single cross-sectional survey to gather the data needed.

A 2019 systematic review of the COC for T2DM in 28 LMICs, including Bangladesh, reported an unmet need for care of 77%, with higher income countries, being female, older age, elevated body mass index, higher educational attainment and higher household wealth associated with lower unmet care needs.^18^ A review of COC for HTN in 44 LMICs found an unmet care need of 90%.^19^ Previous studies on the COC for T2DM in Bangladesh reported screening at 67%,^20^ diagnosis at 31%–47%, treatment at 25%–36% and control at 5%–15%.^8,14,20–22^ The COC for HTN in Bangladesh using the nationally representative BDHS from 2022^8^ reported levels of diagnosis for women at 57%, treatment at 47% and control at 19%, and those for men were 49%, 40% and 20%, respectively.

Studies have also shown that the COC varies within subnational geographies. For example, a 2023 study from India found that most of the variability of the COC was *within* states and not between states,^23^ highlighting how nationally representative averages can both underestimate and overestimate the care gaps. We therefore aimed to define COC for the diagnosis and management of T2DM and HTN at a rural subdistrict level within Faridpur District, Bangladesh. Mapping these subdistrict differences may elucidate locally relevant barriers and facilitators of quality care to help create more targeted and effective interventions.

## Methods

### Study design

We conducted a secondary analysis of two random population cross-sectional surveys conducted in 2021 and 2022, as part of the D:Clare cluster randomised controlled trial (RCT) conducted in Alfadanga Upazila (subdistrict), Faridpur District, Bangladesh.^24^ D:Clare investigated the effect of a community-based participatory learning and action intervention aimed at reducing intermediate hyperglycaemia and T2DM by prompting community actions to tackle NCD risk factors, and the protocol and main trial results were published previously (registration: ISRCTN42219712).^24–26^ Given the relatively short time period between the two surveys, the independent random samples and small absolute changes in T2DM and HTN prevalence (online supplemental appendix 1^26^), we chose to pool the surveys to increase the population available for analysis. Both surveys took place during periods of low COVID-19 transmission without government restrictions, with the vaccination programme launched during the first survey.

### Setting

Alfadanga Upazila has an estimated population of 120 000, subdivided into six unions.^27^ The population is primarily Bengali and 90% Muslim and is mainly reliant on an agricultural economy. The Padma River and other large rivers run through the upazila and are prone to flooding, meaning travel and healthcare access are sometimes hindered.

The healthcare system in Bangladesh is pluralistic, with care provided by government, private, non-governmental organisations and donor agencies,^28^ with high out-of-pocket expenditure (73.0% of total national health expenditure),^29^ and universal health coverage of 52% in 2021.^30^ In Faridpur, previous studies have reported a lack of health service equipment and staff training for NCD care outside of upazila health complexes^31,32^ and barriers to care including inconvenient opening hours, high costs and long waiting times.^32^

In Alfadanga, a publicly funded Upazila Health Complex is located near Alfadanga town in the centre of the upazila, providing primary level care with basic inpatient capacity. The Bangladesh Diabetes Association (BADAS) runs a not-for-profit outpatient diabetes clinic south of Alfadanga Town and a diabetes hospital located in Faridpur City in the neighbouring upazila. Primary healthcare is provided by the government at union-level facilities, and both public and private providers function at the village level (eg, pharmacies, community health centres and informal village doctors).

### Participant selection

The D:Clare trial split the 6 unions into 12 clusters of approximately 10 000 inhabitants each, with a population census done by the research team in January 2021 forming the sampling frame.^24^ Within each study cluster, 2–5 villages were purposely chosen to achieve a total of 800–1000 households, based on the following criteria: not a major trading or administrative centre, minimum of 50 households and not on a border with another cluster. Households were then sampled using simple random sampling, and within households, a single eligible individual was selected using simple random sampling. Eligible individuals were permanent non-pregnant residents aged ≥30 years. A total of 143 individuals from each cluster were selected (n=1716) for each survey, using the same process for the first survey in 2021 (baseline) and the second survey in 2022 (endline). For this study, we included all individuals from the baseline and only participants in the control clusters from the endline survey to avoid measuring any effect from the intervention.

### Data collection

The surveys were administered by trained fieldworkers in the participants’ homes.^24^ It consisted of detailed information on socio-demographic characteristics, lifestyle and behavioural risk factors, NCD diagnoses, care seeking and treatment, adapted from the WHO STEPwise tool and 2011 Bangladesh DHS. Given that the D:Clare trial focused on T2DM, the questionnaire included additional questions on T2DM screening, which were not asked for HTN. At the end of the survey, a ‘global positioning system’ (GPS) measurement of the respondents’ home location was recorded.

Anthropometric data were collected using a standardised protocol on the same day as the survey.^24^ For those without a prior T2DM diagnosis, participants were informed the day before to fast overnight and present in the morning for a capillary blood glucose by fingerprick followed by a 2-hour oral glucose tolerance test (OGTT, ie, 75 g oral glucose load in 250 mL of water followed by a 2-hour postprandial repeat capillary blood test). Among those who reported a prior diagnosis of T2DM, a random blood glucose measurement was taken in the morning. Blood pressure measurements were taken two times, with a 5 min interval between measurements, on a non-clothed upper arm while seated. The blood pressure was then taken as the average between the two readings.

### Defining the cascade of care

The variables used for the COC for T2DM and HTN are summarised in table 1. For T2DM, individuals were defined as having diabetes if they either self-reported a diagnosis by a healthcare professional or they had a fasting glucose ≥7 mmol/L or OGTT ≥11.1 mmol/L, as per WHO criteria.^33^ For T2DM, the COC steps included were self-reported screening for T2DM (screened), self-reported T2DM diagnosis made by health professionals (diagnosed), self-reported use of tablets/pills or insulin injections for T2DM (treated), and a fasting blood glucose <7 mmol/L or random blood glucose <11.1 mmol/L among those with self-reported diabetes (controlled). While adequate control in clinical practice is normally determined by a series of blood glucose measurements over a period or a haemoglobin A1c (HbA1c) value,^33^ we only had a single point measurement. We therefore used the threshold for adequate control as being a random or fasting blood glucose level below the threshold used to diagnose diabetes, in-line with previous studies.^20,23^

**Table 1:** Definitions of the main variables for cascades of care for T2DM and HTN

Term	Denominator	Definition
Person living with diabetes	Analytic sample	Answered ‘yes’ to the question ‘Have you ever been told you have diabetes?’ and for this to have been done by a health professional, OR meeting WHO criteria for T2DM from blood testing (fasting ≥7 mmol/L and/or 2-hour oral glucose tolerance test ≥11.1 mmol/L)
Screened	Person living with diabetes	Answered ’yes’ to the question ‘Have you ever had your blood sugar measured before today?’ with any provider considered eligible (eg, in routine care or during outreach campaigns)
Diagnosed	Person living with diabetes	Answered ‘yes’ to the question ‘Have you ever been told you have diabetes?’ and for this to have been done by a health professional
Treated	Person living with diabetes	Answered ‘yes, currently’ to either of the questions ‘Are you currently or have you ever received drugs (pills or tablets) for your diabetes?’ or ‘Are you currently or have you ever received insulin (ie injections) for your diabetes?’
Controlled	Person living with diabetes	Person living with diabetes with a single measurement below the WHO diagnostic criteria, that is, a fasting glucose <7.0 or random glucose of <11.1 mmol/L
Person living with hypertension	Analytic sample	Answered ‘yes’ to the question ‘Have you ever been told you have raised blood pressure (hypertension)?’ and for this to have been done by a health professional, or having an average systolic blood pressure of ≥140 mmHg or diastolic blood pressure of ≥90 mm Hg from two measurements
Diagnosed	Person living with hypertension	Answered ‘yes’ to the question ‘Have you ever been told you have raised blood pressure (hypertension)?’ and for this to have been done by a health professional
Treated	Person living with hypertension	Answered ‘yes, currently’ to the question ‘Are you now receiving or have you ever received a drug for high blood pressure or hypertension from a doctor?’
Controlled	Person living with hypertension	Person living with hypertension with blood pressure average from two measurements below the diagnostic criteria, that is, systolic blood pressure of <140 mmHg and diastolic blood pressure of <90 mmHg

HTN was defined as having a self-reported prior diagnosis by a healthcare professional or having an average systolic blood pressure ≥140 mmHg or a diastolic blood pressure ≥90 mmHg when averaged over two readings, in line with the WHO criteria.^2,34^ Those who self-reported taking tablets for HTN were defined as ‘treated’, and those with a self-reported prior diagnosis who had an average blood pressure below the HTN threshold were deemed ‘controlled’. The ‘screened’ category was not included in our COC for HTN as we did not ask this question in the survey.

Unmet care need was defined as the percentage of people with the condition that did not reach the final category of the care cascade, that is, having good disease control on treatment.^20,35^ The main exposure variable is the study cluster where the participant lived, determined by the GPS coordinate.

### Analysis

Using descriptive statistics, the proportion and 95% CI for each step in the COC (table 1) was calculated first, followed by the overall unmet need for care for T2DM and HTN separately. For multimorbidity, defined as participants who had both T2DM and HTN, whether they were diagnosed or undiagnosed was calculated. A COC was created for each of the 12 study clusters within Alfadanga Upazila, with separate COC for men and women. The clustered care cascades were presented using choropleth maps generated using -*spmap*- in Stata SE18 and heat map tables.^28,36^ No formal statistical analysis was performed to check for differences between groups, and all data processing was done using Stata SE18. Very few participants had missing information and were dropped (ie, taking a complete case analysis approach). Anonymised data are available in a public open-access repository.^37^

### Patient and community involvement

A community advisory committee was established for the main D:Clare trial to facilitate communication between the researcher team and communities, advise on intervention development and implementation approach and help address community concerns about the project. The community advisory committee included healthcare workers, religious leaders and community members. Although they provided input in the overall project, they were not involved in the formulation, analysis or interpretation related to this paper.

## Results

**Figure 1:**
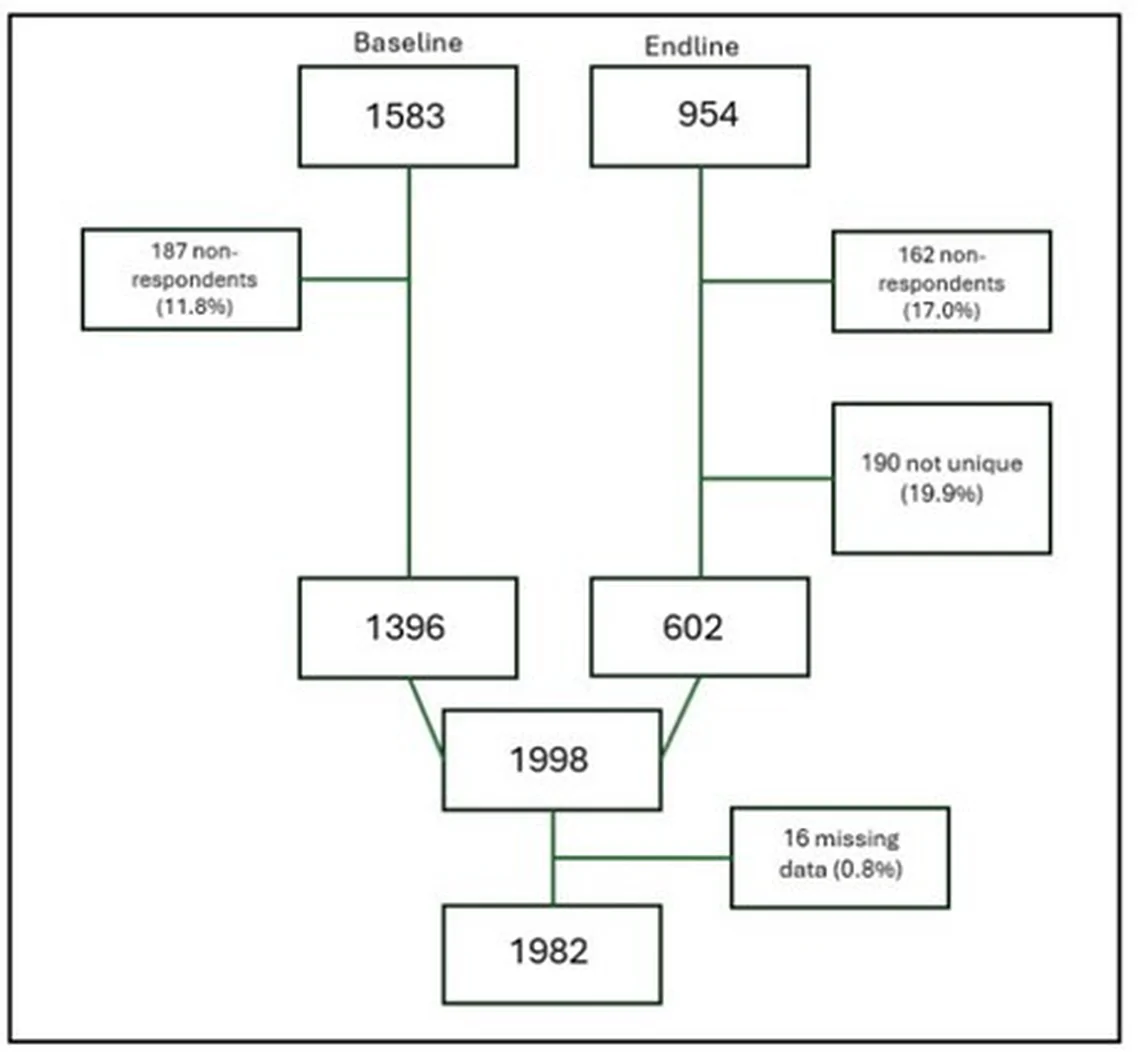
Participant inclusion flow diagram.

Overall, 1396 (89.2% response rate) participants from the baseline survey and 602 from control clusters in the endline survey (83.0% response rate, excluding 190 individuals who were randomly selected for both the baseline and endline surveys) were eligible for inclusion in this study. 16 participants had missing data, giving a final analytic sample of 1982 (figure 1). The mean age was 50.3 years, women made up 62.9% of the participants and the most common occupations were unemployed/housewife for women (96.2%) and manual labour for men (74.1%) (table 2).

**Table 2:** Socio-demographic characteristics of the study participants

		Female (n=1247)	Male (n=735)
Age, years (mean)		49.1	52.4
Highest level of education	No formal education	450 (36.1%)	267 (36.3%)
	Incomplete primary	273 (21.9%)	161 (21.9%)
	Complete primary or higher	524 (42.0%)	307 (41.7%)
Literate*	Yes	653 (52.4%)	401 (54.6%)
Married	Yes	964 (77.3%)	714 (97.1%)
Occupation†	Unemployed/ housework	1200 (96.2%)	127 (17.3%)
	Manual	29 (2.3%)	545 (74.1%)
	Professional	18 (1.4%)	63 (8.6%)
Religion	Islam	1190 (95.4%)	706 (96.1%)
BMI, kg/m^2^‡	Underweight <18.5	121 (9.7%)	109 (14.9%)
	Normal 18.5–22.9	449 (36.0%)	356 (48.5%)
	Overweight 23–24.9	247 (19.8%)	138 (18.8%)
	Obese ≥25	430 (34.5%)	131 (17.9%)
Self-reported current tobacco smoker	Yes	2 (0.2%)	265 (36.1%)

* Literacy was determined by asking participants to read aloud a standardised printed statement.

† Examples of occupation include unemployed/housework (remittance from overseas, retired and housewife), manual (rickshaw driver, farmer, fisherman, day labour and paid domestic work), professional (teacher, healthcare worker, Imam and non-government organisation worker).

‡ BMI was defined according to WPRO 2000.

BMI, body mass index; WPRO 2000, WHO Western Pacific Region 2000.

### Cascade of care for type 2 diabetes mellitus

**Figure 2:**
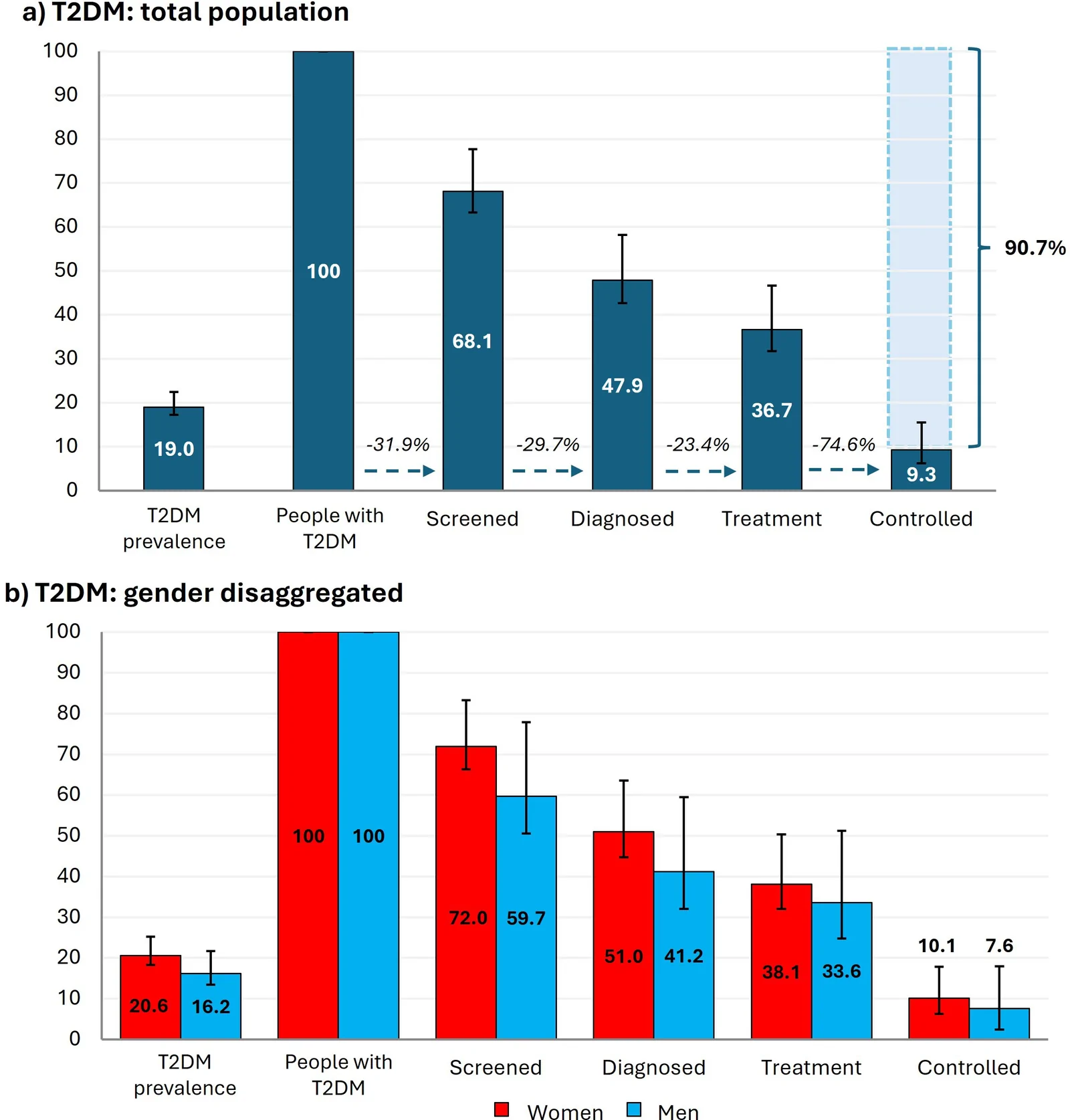
Cascade of care for T2DM in Alfadanga Upazila, Bangladesh. (a) Total population; (b) sex disaggregated. T2DM, type 2 diabetes mellitus.

The prevalence of T2DM was 19.0% (n=376/1982, 95% CI 17.3 to 20.8) and was higher among women than among men (20.6% (95% CI 18.4 to 23.0) vs 16.2% (95% CI 13.6 to 19.1)). Overall, the unmet care need was 90.7% (95% CI 87.1 to 93.2) (figure 2a). The proportion of people who had been diagnosed and were aware of their positive diabetes status was 47.9% (95% CI 42.7 to 53.1), and this was higher among women than among men (51.0% (95% CI 44.7 to 57.2) vs 41.2% (95% CI 32.2 to 50.6) (figure 2b). Women also had higher rates of screening (72.0% (95% CI66.1 to 77.4) vs 59.7% (95% CI 50.3 to 68.6)), treatment (38.1% (95% CI 32.2 to 44.4) vs 33.6% (95% CI 25.2 to 42.8) and control (10.1% (95% CI 6.7 to 14.5) vs 7.6% (95% CI 3.5 to 13.9)) than men, although 95% CIs were wide and overlapping.

Among those who were undiagnosed, 39.8% were classified as having T2DM based on their fasting blood glucose, 32.7% on the OGTT and for 27.6% both. The largest attrition between categories in the care continuum was between treatment and control, in which 74.7% of people who were on drug treatment for T2DM did not have controlled blood glucose according to our criteria (table 1). Among the people with a known diagnosis of T2DM, 169/180 (93.9%) reported having received drug treatment at some point; however, only 139/180 (77.2%) were currently taking tablets or insulin (online supplemental appendix 2).

**Figure 3:**
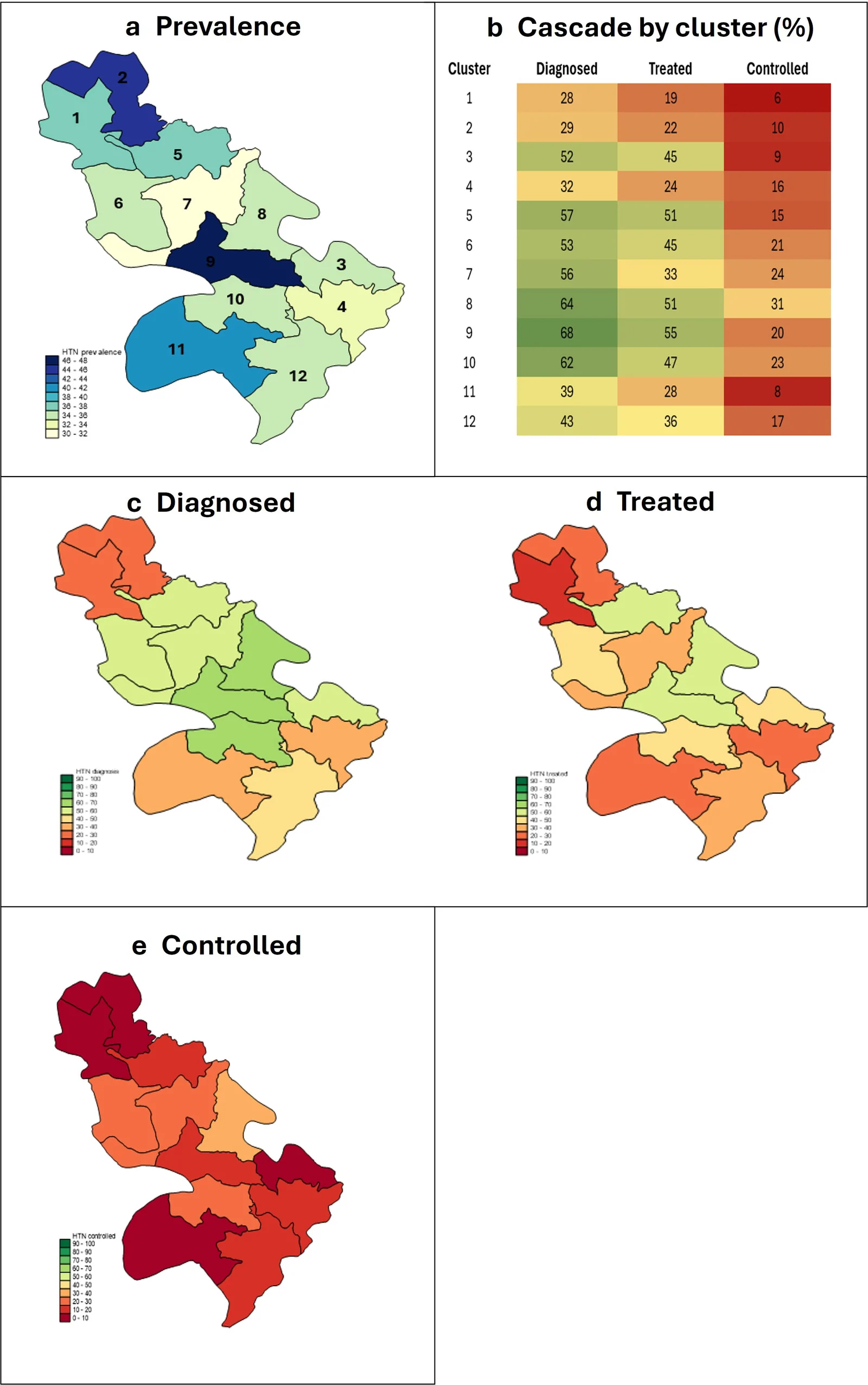
Subregional cascade of care for T2DM in Alfadanga Upazila. (a) Prevalence of T2DM, (b) heat map of the cascade of care by cluster, (c) screening, (d) diagnosis, (e) treatment and (f) control. T2DM, type 2 diabetes mellitus.

In terms of geospatial differences in the COC for T2DM, large differences were observed in T2DM prevalence, ranging from 26.5% (95% CI 20.5 to 33.2) to 12.4% (95% CI 6.9 to 19.9) (figure 3a). Large differences were also seen across the steps in the cascade (figure 3b–f**,** online supplemental appendix 3). Cluster 11 had one of the lowest prevalences of T2DM (12.5%) and the highest level of screening (85.7%) and subsequently of diagnosis (85.7%); however, the unmet need for care was above the average at 92.9%. The lowest levels of screening were in clusters 1 (55.6%), 2 (50.0%), 3 (60.0%) and 7 (56.8%), which also had the lowest levels of diagnostic awareness, with close to two-thirds of people living with diabetes being undiagnosed.

### Cascade of care for hypertension

**Figure 4:**
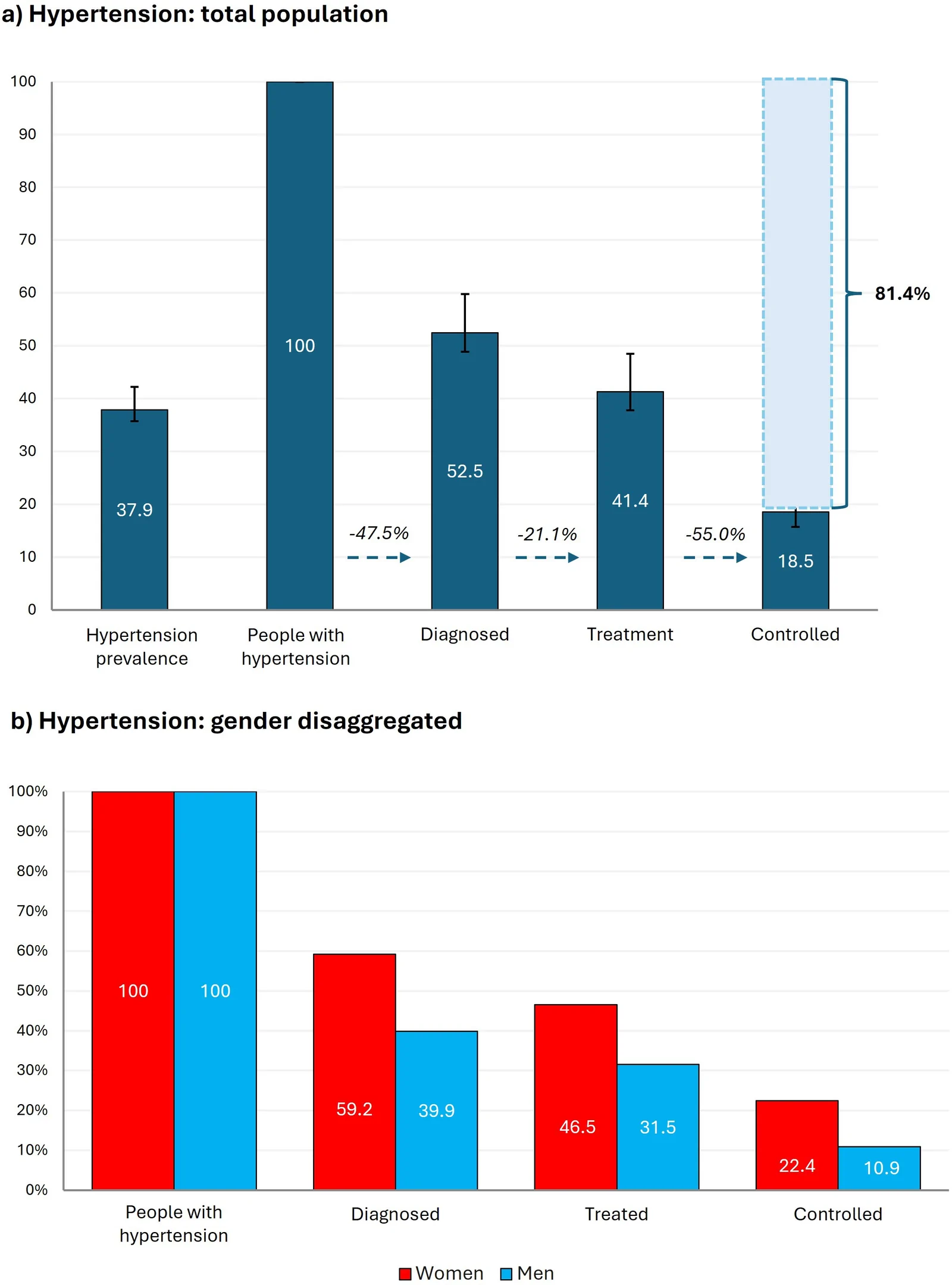
Cascade of care for hypertension in Alfadanga Upazila, Bangladesh. (a) Total population and (b) sex disaggregated.

The population prevalence of HTN was 37.9% (n=752/1982, 95% CI 35.8 to 40.1) and was higher among women than among men (39.7% (95% CI 37.0 to 42.5) vs 35.0% (95% CI 31.5 to 38.5)). The unmet need for care was 81.5% (figure 4a), but differed by sex, with women having a need gap of 77.6% compared with 89.1% in men, and this sex gap was most prominent in those aware of their diagnosis (figure 4b). The largest step change in the cascade was between treatment and control (55.0% attrition), with 41.4% (95% CI 37.8 to 45.0) of people with hypertension on drug treatment, but only 18.5% (95% CI 15.8 to 21.4) achieving control.

**Figure 5:**
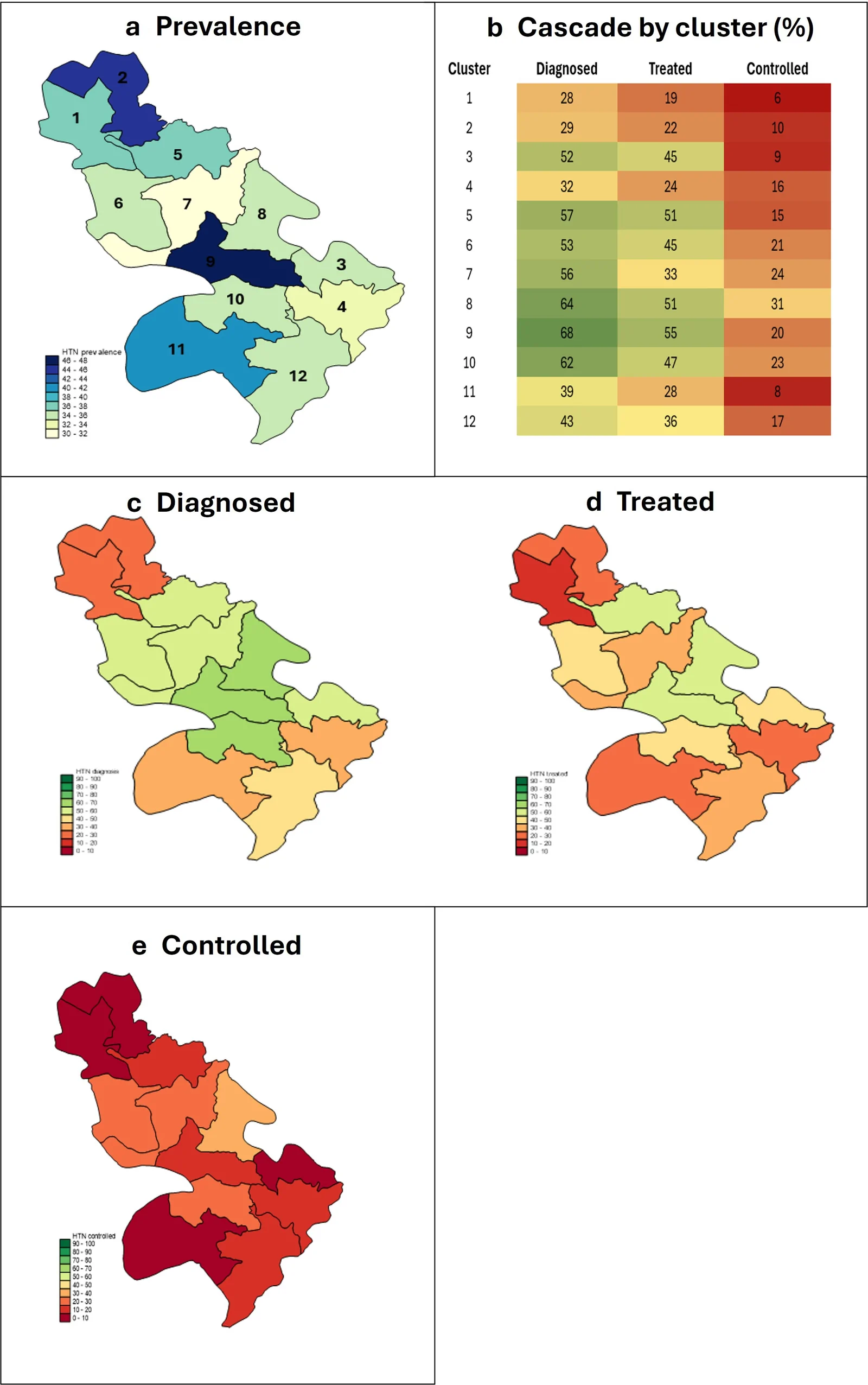
Subregional cascade of care for hypertension in Alfadanga Upazila. (a) Prevalence of T2DM, (b) heat map of the cascade of care by cluster, (c) diagnosis, (d) treatment and (e) control. HTN, hypertension

The HTN prevalence varied between clusters, from 48.5% in cluster 9 to 31.4% in cluster 7 (figure 5**,** online supplemental appendix 4). In terms of the diagnosis of HTN, clusters 8, 9 and 10 had the highest coverage (62.8%–68.0%), compared with clusters 1 and 2 where less than a third of HTN was diagnosed. Subsequently, in clusters 1 and 2, only 6.5% and 10.5% of people with HTN had achieved control of their condition.

### Multimorbidity

T2DM and HTN were found concomitantly in 10.6% (n=210/1982, 95% CI 9.3 to 12.9) of the population. For participants with T2DM, 55.9% (95% CI 50.7 to 60.9) had known or new-found HTN. This proportion was 63.9% (95% CI 56.4 to 70.9) among those with a known prior diagnosis of T2DM.

## Discussion

In this study, we found a 19.0% prevalence of T2DM and 37.9% for HTN, and 10.6% of the population had both conditions concurrently. There were large drop-offs in the COC at the point of screening, diagnosis and control, and 9 out of 10 of those with T2DM and 8 out of 10 people with HTN had unmet care needs. Differences were observed in prevalence and care coverage at the subregional level but also different patterns between men and women, with women having higher rates of T2DM and HTN but slightly lower unmet care needs, although 95% CIs were often wide and overlapping.

Our estimate of the prevalence of T2DM is slightly higher than the most recent BDHS 2022 (17% for women and 15% for men) and is probably partially explained by our eligible population being older (>30 vs >18 years).^8^ Similarly, our population prevalence of 38% HTN was higher than both the national prevalence in 2022 (women 23% and men 17%) and regionally at 32% in 2019.^8,38^ This might reflect a genuine increase but defining HTN from a single measure is sensitive to small differences in diagnostic methods and setting,^36^ and variance between studies in the same setting is often seen. For example, a systematic review of 53 studies spanning the last five decades from Bangladesh found HTN prevalence ranging from 1% to 75%, emphasising this point.^9^

We found a large unmet need for care for both T2DM and HTN with considerable drop-offs in the cascade between each step, offering several opportunities to intervene. Notably, only 68% of the study population reported having been screened for T2DM before, and over half were not aware of their T2DM status. We lacked information on screening coverage for HTN, but given that awareness of their condition was similarly low, understanding where gaps are in HTN screening will be important. Among those diagnosed, treatment coverage was close to the global target of 80% for both conditions, but disease control was low.^39^ This echoes the findings by Islam *et al*,^20^ Khan *et al*^22^ and Anika *et al*^14^ who analysed the 2018 and 2022 nationally representative WHO Steps survey. The low levels of screening and diagnosis are likely due to challenges in human resources training and basic equipment (ie, blood pressure monitor and glucometer) particularly in primary care.^31,32,40,41^ A survey of primary health facilities in Faridpur District found that only upazila health complexes had the necessary equipment and consumables to diagnose T2DM and HTN.^32^

Despite being on treatment, most people living with T2DM or HTN had not achieved control of their condition, although we acknowledge that assessing control with a single random or fasting measurement (based on diagnostic thresholds) may misclassify control compared with HbA1c or repeated measurements. It is uncertain in this study whether this approach underestimated or overestimated the proportion of individuals considered controlled. An analytical model of the COC for HTN in Bangladesh reported that increasing diagnosis awareness by as much as 50% only increased good control by 1.3%–1.8%.^42^ This treatment failure has several drivers, including several health system factors such as the lack of sufficient staff capacity, unreliable medical records for patients, poor availability of prescribed medication and lack of planned follow-ups.^31^ From the individual perspective, there are issues of patient health literacy regarding NCDs and rationing of self-care due to costs. A qualitative study from Faridpur District reported that patients did not prioritise taking medication unless they felt symptomatic,^32^ which normally occurs when blood glucose or blood pressure are at very high levels. This cycle between treatment and poor control can explain why many people on treatment remain uncontrolled.^31,32^

We found a pattern of women having higher prevalence of T2DM and HTN but also higher levels of screening and diagnosis; however, we did not formally test for statistical differences. These results are coherent with the findings of previous studies, and the reasons are multifactorial and complex.^21,38,43,44^ Attrition between the treatment and control cascade categories were similar between sexes however, indicating the sex gap in unmet care needs is the result of increased screening among women. Previous research provides various but sometimes conflicting explanations for this. First, antenatal care in Bangladesh includes T2DM and HTN screening in the national guidance for pregnancy care since 2016,^45^ which means that women of childbearing ages have additional opportunities to be screened for gestational conditions and therefore may be more aware of services and their future risk of diagnosis. Conversely, interviews with people diagnosed with T2DM reported barriers for women to access care requiring a male relative to accompany them to the healthcare facility and provide funds.^32^ Women have also been reported to seek care from informal ‘village doctors’ over formal healthcare facilities.^46^ It will be important to explore this gendered dimension of screening to understand how to improve uptake among men while reducing risks for women.

A subregional variation was noted in the prevalence and unmet needs, and these were not always aligned. Previous studies from Bangladesh, Iran and India have reported wide differences in both NCD prevalence and COC in neighbouring regions^23,47–49^; however, compared with our study that covered approximately 120 000 people, those studies were comparing larger population units of over one million people. For both T2DM and HTN, our maps suggest that disease prevalence is higher in the more urbanised areas, which would be consistent with previous research,^20,50^ but surprisingly, the same pattern is not apparent for care coverage. Although the numbers in this study are too small to determine whether the variation is statistically significant, it raises the hypothesis that hyper-local factors (ie, variations in environmental, health service access and behavioural factors occurring at a village or cluster level) can influence NCD risks and effective healthcare coverage. Online supplemental appendix 5 presents examples of hyper-local factors from this context and how they vary between clusters. One example is farming practices, with some communities heavily engaged in commercial farming of sugar beets, which may lead to differences in sugar consumption from one village to another. Another example from qualitative interviews conducted in Faridpur District is the perception of variable healthcare staff competence in local healthcare facilities,^32^ which could influence the likelihood of seeking care at a particular facility. Our finding of subregional variation, potentially driven by hyper-local factors, highlights the need for reliable local data to more effectively target resources.

This study had several key limitations. First, some data were self-reported. We were unable to triangulate responses against medical records, and this may have led to under-reporting of previous screening and diagnoses. Second, the non-response rate was higher among men, particularly younger men. This may have inflated the population prevalences for T2DM and HTN. Third, using HbA1c to measure long-term blood glucose control would have been a better measure of control; however, it was not logistically possible in the context of this study. Using ambulatory blood pressure cuffs and 24-hour average blood pressure is a more accurate approach for diagnosing HTN, but again, this was not feasible in this study. Finally, we combined data from two cross-sectional surveys that were conducted at different time points, which may have introduced a temporal variation if changes to the prevalence, quality of care or care-seeking occurred.

The existence of subregional variation in disease and care patterns highlights the importance of establishing systems that can monitor in real time the health of communities to improve healthcare responsiveness to population care needs. Addressing the unmet care need of T2DM and HTN and minimising their long-term complications in this setting will need both community and healthcare service focused interventions. Further research should consider how to tailor services that address gendered care-seeking behaviours and take into account local variations in service uptake and quality.

## Supplementary material

10.1136/bmjph-2025-004574 online supplemental file 1

## Data Availability

Data are available in a public, open access repository.

## References

[1] Ong KL, Stafford LK, McLaughlin SA (2023). Global, regional, and national burden of diabetes from 1990 to 2021, with projections of prevalence to 2050: a systematic analysis for the Global Burden of Disease Study 2021. The Lancet.

[2] (2023). Global report on hypertension: the race against a silent killer. https://www.who.int/publications/i/item/9789240081062.

[3] World Health Organization (2014). Global status report on noncommunicable diseases 2014. https://iris.who.int/handle/10665/148114.

[4] Kario K, Okura A, Hoshide S (2024). The WHO Global report 2023 on hypertension warning the emerging hypertension burden in globe and its treatment strategy. Hypertens Res.

[5] (2024). Global diabetes data report 2000 — 2045. https://diabetesatlas.org/data/.

[6] Banerjee A, Hurst J, Fottrell E (2020). Multimorbidity: Not Just for the West. Glob Heart.

[7] Mohiuddin AKA (2019). Diabetes Fact: Bangladesh Perspective. Community and Public Health Nursing.

[8] National Institute of Population Research and Training (NIPORT) and ICF (2024). Bangladesh demographic and, health survey 2022: final report. Dhaka, Bangladesh, and Rockville, Maryland, Usa: NIPORT and ICF. National Institute of Population Research and Training (NIPORT) and ICF. 2024. Bangladesh demographic and health survey 2022: final report. https://preview.dhsprogram.com/pubs/pdf/FR386/FR386.pdf.

[9] Khan N, Rahman M, Mitra D (2019). Prevalence of multimorbidity among Bangladeshi adult population: a nationwide cross-sectional study. BMJ Open.

[10] (1993). The Effect of Intensive Treatment of Diabetes on the Development and Progression of Long-Term Complications in Insulin-Dependent Diabetes Mellitus. N Engl J Med.

[11] Kario K, Mogi M, Hoshide S (2022). Latest hypertension research to inform clinical practice in Asia. Hypertens Res.

[12] Bangladesh, WHO Bangladesh (2018). Multisectoral action plan for prevention and control of noncommunicable diseases 2018-2025: with a three year operational plan.

[13] de Silva A, Varghese C, Amin MR (2023). Non-communicable diseases in South-East Asia: journeying towards the SDG target. *Lancet Reg Health Southeast Asia*.

[14] Anika US, Rafi MA, Hossain MG (2025). Diabetes care cascade in Bangladesh: Identifying gaps and social determinants. Diabetes Res Clin Pract.

[15] Perlman DC, Jordan AE, Nash D (2016). Conceptualizing Care Continua: Lessons from HIV, Hepatitis C Virus, Tuberculosis and Implications for the Development of Improved Care and Prevention Continua. Front Public Health.

[16] Ali MK, Bullard KM, Gregg EW (2014). A cascade of care for diabetes in the United States: visualizing the gaps. Ann Intern Med.

[17] Wang J, Tan F, Wang Z (2024). Understanding Gaps in the Hypertension and Diabetes Care Cascade: Systematic Scoping Review. JMIR Public Health Surveill.

[18] Manne-Goehler J, Geldsetzer P, Agoudavi K (2019). Health system performance for people with diabetes in 28 low- and middle-income countries: A cross-sectional study of nationally representative surveys. PLoS Med.

[19] Geldsetzer P, Manne-Goehler J, Marcus M-E (2019). The state of hypertension care in 44 low-income and middle-income countries: a cross-sectional study of nationally representative individual-level data from 1·1 million adults. *The Lancet*.

[20] Islam MT, Bruce M, Alam K (2023). Cascade of diabetes care in Bangladesh, Bhutan and Nepal: identifying gaps in the screening, diagnosis, treatment and control continuum. Sci Rep.

[21] Khan N, Oldroyd JC, Hossain MB (2022). Awareness, Treatment, and Control of Diabetes in Bangladesh: Evidence from the Bangladesh Demographic and Health Survey 2017/18. Int J Clin Pract.

[22] Rahman MdS, Akter S, Abe SK (2015). Awareness, Treatment, and Control of Diabetes in Bangladesh: A Nationwide Population-Based Study. PLoS ONE.

[23] Varghese JS, Anjana RM, Geldsetzer P (2023). Diabetes diagnosis, treatment, and control in india: results from a national survey of 1.65 million adults aged 18 years and older, 2019-2021. Endocrinology (Including Diabetes Mellitus and Metabolic Disease).

[24] King C, Pires M, Ahmed N (2021). Community participatory learning and action cycle groups to reduce type 2 diabetes in Bangladesh (D:Clare trial): study protocol for a stepped-wedge cluster randomised controlled trial. Trials.

[25] King C, Pires M, Ahmed N (2022). Community participatory learning and action cycle groups to reduce type 2 diabetes in bangladesh (dclare): an updated study protocol for a parallel arm cluster randomised controlled trial. *In Review*.

[26] Fottrell E, Kuddus A, Morrison J (2025). A cluster randomised controlled trial of community groups using Participatory Learning and Action to prevent and control diabetes and intermediate hyperglycaemia in rural Bangladesh. *PLOS Glob Public Health*.

[27] Bangladesh Bureau of Statistics (2022). Population and housing census.

[28] Pacific WHORO for the W (2015). Bangladesh health system review. Health Syst Transit.

[29] World Health Organisation (2025). Health financing: out-of-pocket expenditure as percentage of current health expenditure (CHE) (%) data by country. https://apps.who.int/gho/data/node.searo.GHEDOOPSCHESHA2011?lang=en.

[30] World Health Organisation Service coverage indicators: index of service coverage data by country. https://apps.who.int/gho/data/node.searo.INDEXOFESSENTIALSERVICECOVERAGE?lang=en.

[31] Huque R, Nasreen S, Ahmed F (2018). Integrating a diabetes and hypertension case management package within primary health care: a mixed methods feasibility study in Bangladesh. BMC Health Serv Res.

[32] Jennings HM, Morrison J, Akter K (2021). Care-seeking and managing diabetes in rural Bangladesh: a mixed methods study. BMC Public Health.

[33] World Health Organization, International Diabetes Federation (2006). Definition and diagnosis of diabetes mellitus and intermediate hyperglycaemia: report of a WHO/IDF consultation. https://iris.who.int/handle/10665/43588.

[34] Riley L, Guthold R, Cowan M (2016). The World Health Organization STEPwise Approach to Noncommunicable Disease Risk-Factor Surveillance: Methods, Challenges, and Opportunities. Am J Public Health.

[35] Yan LD, Hanvoravongchai P, Aekplakorn W (2020). Universal coverage but unmet need: National and regional estimates of attrition across the diabetes care continuum in Thailand. PLoS One.

[36] Mousavi SS, Reyna MA, Clifford GD (2023). A survey on blood pressure measurement technologies: addressing potential sources of bias. https://arxiv.org/abs/2306.08451v3.

[37] Fottrell, Edward; King, Carina; Azad, Kishwar (2025). The bangladesh diabetes: community-led awareness, response and evaluation (DClare) cluster randomised controlled trial data.

[38] Khan MdN, Oldroyd JC, Chowdhury EK (2021). Prevalence, awareness, treatment, and control of hypertension in Bangladesh: Findings from National Demographic and Health Survey, 2017–2018. J of Clinical Hypertension.

[39] (2023). First-ever global coverage targets for diabetes adopted at the 75th world health assembly. https://www.who.int/news-room/feature-stories/detail/first-ever-global-coverage-targets-for-diabetes-adopted-at-the-75-th-world-health-assembly.

[40] Kabir A, Karim MN, Billah B (2021). Primary healthcare system readiness to prevent and manage non-communicable diseases in Bangladesh: a mixed-method study protocol. BMJ Open.

[41] Sameh ME, Sparkes SP, Barroy H (2024). The path to universal health coverage in Bangladesh: bridging the gap of human resources for health. https://documents.worldbank.org/en/publication/documents-reports/documentdetail/686591467986284082/The-path-to-universal-health-coverage-in-Bangladesh-bridging-the-gap-of-human-resources-for-health.

[42] Datta BK, Ansa BE, Husain MJ (2022). An analytical model of population level uncontrolled hypertension management: a care cascade approach. J Hum Hypertens.

[43] Chowdhury MAB, Islam M, Rahman J (2022). Diabetes among adults in Bangladesh: changes in prevalence and risk factors between two cross-sectional surveys. BMJ Open.

[44] Babagoli MA, Chen YH, Chakma N (2023). Association of socio-demographic characteristics with hypertension awareness, treatment, and control in Bangladesh. J Hum Hypertens.

[45] Bear AP, Bennett WL, Katz J (2023). Self-reported diabetes or hypertension diagnoses and antenatal care among child-bearing women in rural Bangladesh: A cross-sectional study. *PLOS Glob Public Health*.

[46] Shah B, Krishnan N, Kodish SR (2020). Applying the Three Delays Model to understand emergency care seeking and delivery in rural Bangladesh: a qualitative study. BMJ Open.

[47] Roy PK, Khan MHR, Akter T (2019). Exploring socio-demographic-and geographical-variations in prevalence of diabetes and hypertension in Bangladesh: Bayesian spatial analysis of national health survey data. Spat Spatiotemporal Epidemiol.

[48] Azadnajafabad S, Ahmadi N, Rezaei N (2023). Evaluation of the diabetes care cascade and compliance with WHO global coverage targets in Iran based on STEPS survey 2021. Sci Rep.

[49] Prenissl J, Jaacks LM, Mohan V (2019). Variation in health system performance for managing diabetes among states in India: a cross-sectional study of individuals aged 15 to 49 years. BMC Med.

[50] Chowdhury MZI, Rahman M, Akter T (2020). Hypertension prevalence and its trend in Bangladesh: evidence from a systematic review and meta-analysis. Clin Hypertens.

[51] Pacific WHORO for the W (2000). The Asia-pacific perspective: redefining obesity and its treatment. https://iris.who.int/handle/10665/206936.

