# Supplementary material for: Cascade of care for type 2 diabetes mellitus and hypertension in rural Bangladesh: sub-regional mapping of gaps in care
Source: BMJ Public Health. 2026 Sep 11;4(3):e004574. doi: 10.1136/bmjph-2025-004574 (PMC13583752; doi:10.1136/bmjph-2025-004574)
Supplement: Supplementary file 1 [file bmjph-4-3-s001.docx]

**Appendix 1: Comparison of T2DM and hypertension prevalence between survey 1 and 2**

The table presents the prevalence of T2DM and hypertension from the control arm of the DClare trial^1^, comparing the baseline and endline survey values using a chi2 test adjusted for trial cluster.

|  | **Survey 1**  **% (95% CI)** | **Survey 2**  **% (95% CI)** | **p-value** |
| --- | --- | --- | --- |
| T2DM | 20.9% (17.9, 24.1) | 21.3% (18.1, 24.8) | 0.872 |
| Hypertension | 39.7% (36.0, 43.4) | 35.3% (31.5, 39.3) | 0.112 |

*Reference:*

1. Fottrell E, Kuddus A, Morrison J, Nahar T, King C, et al. (2025) A cluster randomised controlled trial of community groups using Participatory Learning and Action to prevent and control diabetes and intermediate hyperglycaemia in rural Bangladesh. PLOS Global Public Health 5(8): e0005049. <https://doi.org/10.1371/journal.pgph.0005049>

**Appendix 2: Detail of treatment amongst those diagnosed with T2DM**

**Appendix 3: Percentage estimates and 95% confidence intervals for prevalence of T2DM and cascade of care for each cluster**

| Cluster  (N) | T2DM Prevalence | Screened | Diagnosed | Treatment | Control |
| --- | --- | --- | --- | --- | --- |
| **1**  (122) | 14.8  (9.5, 22.3) | 55.6  (30.8, 78.5) | 33.3  (13.3, 59.0) | 22.2  (6.4, 47.6) | 5.6  (0.1, 27.3) |
| **2**  (124) | 19.4  (12.8, 27.4) | 50.0  (29.1, 70.9) | 33.3  (15.6, 55.3) | 25.0  (7.7, 46.7) | 4.2  (0.1, 21.1) |
| **3**  (121) | 16.5  (10.4, 24.4) | 60.0  (36.1, 80.9) | 30.0  (11.9, 54.3) | 20.0  (5.7, 43.7) | 5.0  (0.1, 24.9) |
| **4**  (110) | 15.6  (9.4, 23.8) | 76.5  (50.1, 93.2) | 52.9  (27.8, 77.0) | 47.1  (23.0, 72.2) | 11.8  (1.5, 36.4) |
| **5**  (219) | 23.7  (18.3, 29.9) | 65.4  (50.9, 78.0) | 46.2  (32.2, 60.5) | 38.5  (25.3, 53.0) | 9.6  (3.2, 21.0) |
| **6**  (224) | 18.8  (13.9, 24.5) | 61.9  (45.6, 76.4) | 50.0  (34.2, 65.8) | 38.1  (23.6, 54.4) | 11.9  (4.0, 25.6) |
| **7**  (207) | 17.9  (12.9, 23.8) | 56.8  (39.5, 72.9) | 29.7  (15.9, 47.0) | 24.3  (11.8, 41.2) | 5.4  (0.7, 18.2) |
| **8**  (216) | 18.5  (13.6, 24.4) | 75.0  (58.8, 87.3) | 55.0  (38.5, 70.7) | 35.0  (20.6, 51.7) | 15.0  (5.7, 29.8) |
| **9**  (200) | 26.5  (20.5, 33.2) | 77.4  (63.8, 87.7) | 56.6  (42.3, 70.2) | 49.1  (35.1, 63.2) | 7.5  (2.1, 18.2) |
| **10**  (214) | 21.0  (15.8, 27.1) | 80.0  (65.4, 90.4) | 57.8  (42.2, 72.3) | 44.4  (29.6, 60.0) | 13.3  (5.1, 26.8) |
| **11**  (112) | 12.5  (7.0, 20.1) | 85.7  (57.2, 98.2) | 85.7  (57.2, 98.2) | 57.1  (28.9, 82.3) | 7.1  (0.2, 33.9) |
| **12**  (113) | 12.4  (6.9, 19.9) | 64.3  (35.1, 87.2) | 35.7  (12.8, 64.9) | 21.4  (4.7, 50.8) | 7.1  (0.2, 33.9) |

**Appendix 4: Percentage estimates and 95% confidence intervals for prevalence of hypertension and cascade of care for each cluster**

| Cluster  (N) | HTN prevalence | Diagnosis | Treatment | Control |
| --- | --- | --- | --- | --- |
| **1**  (122) | 37.7  (29.1, 46.9) | 28.3  (16.0, 43.5) | 19.6  (9.4, 33.9) | 6.5  (1.4, 17.9) |
| **2**  (124) | 46.0  (37.0, 55.1) | 29.8  (18.4, 43.4) | 22.8  (12.7, 35.8) | 10.5  4.0, 21.5) |
| **3**  (121) | 36.4  (27.8, 45.6) | 52.3  (36.7, 67.5) | 45.5  (30.4, 61.2) | 9.1  (2.5, 21.7) |
| **4**  (110) | 33.6  (24.9, 43.3) | 32.4  (18.0, 49.8) | 24.3  (11.8, 41.2) | 16.2  (6.2, 32.0) |
| **5**  (219) | 37.9  (31.4, 44.7) | 57.8  (46.5, 68.8) | 51.8  (40.6, 62.9) | 15.7  (8.6, 25.3) |
| **6**  (224) | 36.6  (30.3, 43.3) | 53.7  (42.3, 64.7) | 45.1  (34.1, 56.5) | 22  (13.6, 32.5) |
| **7**  (207) | 31.4  (25.1, 38.2) | 56.9  (44.0, 69.2) | 33.8  (22.6, 46.6) | 24.6  (14.8, 36.9) |
| **8**  (216) | 35.2  (28.8, 42.0) | 64.5  (52.7, 75.1) | 51.3  (40.0, 63.0) | 31.6  (21.4, 43.3) |
| **9**  (200) | 48.5  (41.1, 55.7) | 68  (57.8, 77.1) | 55.7  (45.2, 65.8) | 20.6  (13.1, 30.0) |
| **10**  (214) | 36.4  (30.0, 43.3) | 62.8  (51.1, 73.5) | 47.4  (36.0, 59.1) | 23.1  (14.3, 34.0) |
| **11**  (112) | 41.1  (31.9, 50.8) | 39.1  (25.1, 54.6) | 28.2  (16.0, 43.5) | 8.7  (2.4, 20.8) |
| **12**  (113) | 36.3  (27.4, 45.9) | 43.9  (28.5, 60.3) | 36.6  (22.1, 53.1) | 17.1  (7.2, 32.1) |

**Appendix 5: Description of Union’s within Alfadanga.** *Note: each union has two clusters, and all are considered rural areas.*

| **Clusters** | **Road access** | **Flood risk** | **Predominant crops** | **Main occupations** | **Healthcare access** |
| --- | --- | --- | --- | --- | --- |
| 3 + 4 | Road access and transportation is quite good, with most roads are paved or brick-laid. Access to waterways is also available. Walking is convenient in most parts. | Not considered prone to flooding or other natural disasters | Staple crops are similar across all Unions, with rice, onion, onion seeds and jute being the most common.  Other crops grown are pulses (lentil), mustard, garlic, sugarcane and seasonal vegetables | High employment, mainly day labourers  and people who work on ships. | Good access to primary healthcare |
| 1 + 2 | Road access and transportation is mostly good. However, rickshaws and bicycle vans are not available for transportation in the river erosion areas, where people depend on boats. | Flooding is not common, but river erosion happens during heavy rainfall between May and July as the river overflows, resulting in displacement of people who seek shelter elsewhere. |  | Lower employment, mainly day labourers and farming, including for women. | Good access to primary healthcare, but there are  facilities in the areas affected by river erosion. |
| 11 + 12 |  |  |  | High employment-mainly day labourers and people who work on ships. |  |
| 5 + 6 | Road access and transportation is quite good, with most roads are paved or brick-laid. Access to waterways is also available. Walking is convenient in most parts. | Not considered prone to flooding or other natural disasters |  | Lower employment, mainly day labourers and farming, including for women. | Good access to primary healthcare, and there is good access to a specialized diabetes hospital. |
| 7 + 8 |  |  |  | Mainly day labour and farming, but also those with office jobs. | Good access to primary healthcare |
| 9 + 10 |  |  |  | High employment, mainly day labourers and people who work on ships. |  |
